# Pain Candidate Genes 5-HTTLPR and COMT Affect Anxiety and Mood in Japanese Ballet Dancers: A Cross-Sectional and Longitudinal Study

**DOI:** 10.3390/sports12110293

**Published:** 2024-10-25

**Authors:** Kanaka Yatabe, Kohei Ashikaga, Ryota Muroi, Shu Somemura, Masahiro Takemoto, Kazuo Yudoh, Hisao Miyano, Hiroto Fujiya

**Affiliations:** 1 Department of Sports Medicine, St. Marianna University School of Medicine, 2-16-1 Sugao, Miyamae-ku, Kawasaki 216-8511, Japan; 2 Department of Orthopaedic Surgery, St. Marianna University School of Medicine, 2-16-1 Sugao, Miyamae-ku, Kawasaki 216-8511, Japan; 3 Department of Frontier Medicine, Institute of Medical Science, St. Marianna University School of Medicine, Kawasaki 216-8511, Japan; 4 Department of Cognitive and Information Sciences, Faculty of Letters, Chiba University, 1-33 Yayoi-cho, Inage-ku, Chiba 263-8522, Japan

**Keywords:** genetics, pain, psychological tests, mental processes, dancing

## Abstract

The balance of mental, physical, and technical aspects is essential in improving ballet performance. Ballet dancers’ emotional and behavioral characteristics vary, even under identical stress conditions. This study aimed to investigate the association between the pain candidate genes 5-HTTLPR and COMT and anxiety in Japanese ballet dancers. Participants were 18 youth elite ballet students with professional aspirations (Y-Elite) and 16 dancers in a professional ballet company (Pro). We administered psychological questionnaires, the State-Trait Anxiety Inventory (STAI) and Brunel Mood Scale (BRUMS), to participants under the following four different stress conditions: standard practice day, cast decision day, rehearsal day, and one week before competition day. In addition, the genotypes of 5-HTTLPR and COMT Val158Met were examined. The distribution of 5-HTTLPR was not different between Y-Elite and Pro dancers, although one of the COMT genotypes was different. Y-Elite dancers had higher trait anxiety scores than Pro dancers for these genotypes before competition (ps < 0.03), although no significant association was observed between both genotypes and scores on the STAI across conditions. Their moods were significantly different through the four conditions (*p* < 0.004). Pro dancers’ moods were also more stable than those of the Y-Elite dancers in the presence of pain. The results indicate that 5-HTTLPR and COMT play a crucial role in dancers’ anxiety and mood during pain (ps < 0.05). Pro dancers are probably predicted by their lower neuroticism and mood scores and their better adaptation to stress than Y-Elite dancers. The 5-HTTLPR and COMT genes may be influencing the sensitivity to the environment. Youth elite ballet dancers need to understand the relationship between pain and physical activity from an early stage.

## 1. Introduction

Balancing the mental, physical, and technical aspects of preparation is critical in improving ballet performance. Although the physical and technical aspects are easy to evaluate objectively [1,2,3,4], the mental elements vary considerably across individuals because they are affected by unconscious stress reactions. The effects of mental stress, presumed to be related to actual performance, are unavoidable. Moreover, they are difficult to elicit and, thus, extremely difficult to evaluate objectively. Each emotional and behavioral characteristic is entirely different even under the same stress conditions [5].

Ballet dancers participate in one or more competitions each year and spend the rest of the months practicing almost every day. They must continue to perform despite different possibilities or stress factors. Their schedules usually include a standard practice period, a period before the stage performance to determine the cast, memorizing the choreography, and completing a series of movements and flows. Their schedules also include a period of repeated rehearsals [6,7,8,9,10] where the dancer memorizes the choreography, which is when they begin to face increased psychological stress. This stress is also often conditioned on acquiring the role in the cast and the requirements for the assigned role. Additionally, the pressure of learning choreography and receiving instructions from a special teacher weighs heavily on the dancer [11,12,13]. The pre-performance rehearsal period requires substantial practice time to complete the choreography. As a result, this is a period characterized by high physical stress, which leads to increased mental and physical fatigue [12,13,14]. From the beginning of stage choreography, ballet dancers’ mental stress increases during casting. As the competition nears, they experience heavy physical stress [12,13]. The dancers express beauty and art through performance, which requires imaginative physical expression and ability. Additionally, performance must continue despite the potential stressors [14].

Performing artists suffer from performance anxiety. Moreover, the incidence of depression was very high due to their occupation, their need to survive within the profession, and their extraordinary lifestyle stress [15]. In sports, prolonged and unmanaged pain can be a stressor that adversely affects athletes mentally and physiologically, often leading to the development of chronic disorders, and creates a negative cycle of pain and stress accumulation. Psychological conditions related to personality traits and moods can also be affected at the level of gene polymorphisms, especially for candidate genes influencing the experiences of pain (such as 5-HTT and COMT), which is associated with quality of life (QOL) [16]. The 5-HTT and COMT genes have been subjects of interest in the context of depression and anxiety. When pain becomes chronic, it causes plasticity changes not only in the peripheral nerves and spinal cord but also in the brain, leading to depression, anxiety disorders, and even sleep disorders, thus also reducing QOL.

The serotonin (5-HTT) gene is known to be potentially associated with anxiety-related traits [17]. The serotonin transporter and arginine vasopressin receptors are associated with a creative dance performance [18]. A short (S) allele is said to be more sensitive to stress when compared to a long (l) allele in the promoter region of the serotonin (5-HTT) gene polymorphic region (called 5-HTTLPR) [19]. The S allele can occur in up to 70–80% of individuals in some East Asian populations compared with the 40–45% occurrence in the typical European sample, wherein individuals are S carriers of the 5-HTT genotype [20,21]. The 5-HT gene-linked polymorphism region (5-HTTLPR) is a repeat polymorphism in the promoter region of the SLC6A4 (encoding 5-HTT) gene, whose alleles contain 14 (short) and 16 (long) base pairs of sequence repeats. The short mutant allele S is supported by lower transcriptional activity and enhanced anxiety in the SLC6A4 gene than the long mutant allele (l) [17].

In our previous study, we investigated whether the serotonin transporter gene polymorphism region (5-HTTLPR) could predict the anxiety and mood status of Japanese ballet dancers [22]. Although not predictable in dancers who belong to a professional ballet company and perform professionally (Pro), we were able to group academic students who are specializing in ballet who have not yet found a job (Y-Elite) by the 5-HTTLPR gene polymorphism and predict their mood states, including depression and anxiety. Only by examining the youth elite ballet dancers’ anxiety could we predict their mood status. The results revealed that the differences in circumstances between professional and vocational (Pro) dancers and youth elite student (Y-Elite) dancers seemed to have much stronger effects on the dancers’ mental states than the 5-HTTLPR genotype [22].

Next, the trait anxiety score should optimize the prediction accuracy by combining data from other genotypes such as noradrenaline and dopamine-related gene polymorphisms [23]. For a comprehensive study, we also analyzed the relationship of the trait anxiety score with the Catechol-O-methyltransferase (COMT) gene. Individuals with the Val/Val-type COMT gene (G allele carriers) have strong COMT activity, because of which dopamine is metabolized, endorphins are released, and pain sensitivity is reduced [24]. However, individuals with the Met/Met-type COMT gene (A allele carriers) are expected to experience hypofunction of the μ-opioid system, hyperalgesia, and negative moods [25]. Additionally, heterozygous Val/Met allele carriers exhibit an intermediate enzyme activity. The findings suggest an association between the COMT polymorphism and specific expressions of anxiety among women in the Australian population [26].

In humans, the COMT protein is coded by the COMT gene. This gene is associated with allelic variants. The COMT Val158Met polymorphism on amygdala action also has multifaceted effects on emotional processing. It is increasingly recognized that allelic variants of the COMT gene also appear to affect the interaction between the prefrontal cortex and limbic system, which are associated with emotional processing. Those with Val/Val genes tend to be more outgoing, more novelty-seeking, and less neurotic than those with the Met/Met allele [25,26]. The COMT gene has also been associated with multiple types of pain; the COMT Val158Met genotype influences μ-opioid neurotransmitter responses to pain stressors [27]. The theory of neurotransmitters and neuroplasticity has the most robust evidence of a relationship between the COMT gene and pain and negative moods, which is expected. In summary, both the 5-HTT and COMT genes play a role in mood regulation.

As described above, the 5HTTLPR gene is strongly associated with anxiety sensitivity and anxiety responses, and the COMT gene is strongly associated with the loss of mood and pain. We have continued testing in ballet dancers under different stress conditions to determine the relationship between genetic testing and the State-Trait Anxiety Inventory (STAI), a measure of anxious mood [28,29,30], and the Brunel Mood Scale (BRUMS), a measure of mood change [31,32,33,34,35]. The BRUMS questionnaire has been used extensively in the domain of sport psychology to investigate the antecedents, correlates, and behavioral consequences of moods. In particular, it has been used to understand the effects of moods on the performance and psychological well-being of athletes and exercisers [36]. In our other research on youth elite students’ ballet, a strong relationship was indicated between the STAI and BRUMS on usual practice days before an intervention, the cast decision day in the early stages of the intervention, and the competition day [13].

In this study, we analyzed the answers to these psychological questionnaires (the STAI and BRUMS) and whether the gene polymorphisms of ballet dancers, specifically those with pain, could indicate anxiety and mood states. Therefore, our study aimed to investigate the relationship between polymorphisms in pain candidate genes (5-HTT and COMT) and the ballet dancers’ anxiety and mood throughout a usual practice day and performance day. In addition, we examined whether the different ballet environments in which professional and vocational dancers (Pro group) and youth elite ballet students with professional aspirations (Y-Elite group) perform ballet affect the change in mood states and influences their stress conditions.

## 2. Materials and Methods

### 2.1. Participants

Thirty-four female ballet dancers, including eighteen adolescents (Y-Elite; 18.2 ± 0.4 years) and sixteen professionals (Pro; 29.8 ± 6.0 years), participated in the study. All of the dancers reported experiencing a ballet injury persisting for many years, and about half pre-self-reported feelings of pain.

### 2.2. Procedure

We administered two psychological questionnaires, namely, the BRUMS and subjective pain feeling, to the participants under the following four different stress conditions: standard practice day (usual), cast decision day, rehearsal day, and one week before competition day. In addition, anxiety and mood were evaluated using the STAI in the usual and pre-competition conditions. The 5-HTTLPR and COMT Val158Met genotypes were also examined. DNA was extracted from the oral mucosa, and 5-HTTLPR and COMT were assessed by polymerase chain reaction (PCR) and cross-checked using at least two primer sets. 

This research followed the Helsinki Declaration and was performed with the approval of the St. Marianna University School of Medicine, Bioethics Committee (No. 1844, Gene 80). The study, including all procedures, possible risks, and benefits, was explained to all participants. Those who volunteered to participate submitted written informed consent. DNA samples were collected only from those who agreed and were over 20 years of age. DNA samples were collected over four time conditions and analyzed after the competition.

### 2.3. Questionnaire

#### 2.3.1. BRUMS

The original BRUMS was developed as a shortened version with a 24-item mood scale; it is appropriate for both adolescents and adults [31,32]. Therefore, the items under consideration were screened for adolescent comprehension and reduced to 24 items through factor validity testing before inclusion in the initial list of 42 items. The BRUMS 6-factor model is a standardization of the original questionnaire on simple moods in adolescents and adults [33,34]. Vigor is a positive indicator, while Tension, Depression, Anger, Fatigue, and Confusion are negative indicators. On the BRUMS [31,32], respondents were asked to rate “How are you feeling right now?” Representative feelings, such as Vigor, Tension, Depression, Confusion, Anger, and Fatigue, were expressed on a scale from 0 to 4 (not at all to extreme). A variety of item counts and scales are currently being translated around the world [36]. The Japanese version could not be limited to 24 items due to language and cultural differences. In this study, from the viewpoint of reliability and validity, we used the Japanese version of the BRUMS for adults, which comprises 42 items (7 items for each of the 6 scales) [37,38].

#### 2.3.2. STAI

The STAI is a well-known 40-item instrument that measures temporary and permanent anxiety levels, respectively [28,29]. In this study, we used the Japanese version of the STAI [30]. Participants were asked to rate the frequency of experiencing each of the 10 items. The specific instructions were as follows: “A number of statements which people have used to describe themselves are given below. Please show how you feel normally (trait anxiety) or right now (state anxiety).” Scores ranged from 1 (almost never) to 4 (almost always). 

### 2.4. Experimental Design for Genotyping

DNA samples were taken from the oral mucosa. The yield (ng/μL) and quality (A260/280) of DNA were assessed using a NanoDrop ND-1000 spectrophotometer (Thermo Fisher Scientific Inc., Wilmington, DE, USA). DNA samples were subsequently stored at −20 °C until further use. DNA was extracted from the buccal epithelium in a swab using a QIAamp DNA Micro kit (Qiagen). In addition, the 5-HTTLPR and COMT of each dancer were assessed by PCR. 

#### 2.4.1. 5-HTTLPR

PCR amplification was performed with the 5-HTTLPR primers F/R 5′-TCCTCCGCTTTGGCGCCTCTTCC-3′ and 5′-TGGGGGTTGCAGGGGAGATCCTG-3′ (as described in 47 references, and 1 by Pieper et al. [39]), mixed in a total volume of 20 μL comprising 5–10 ng of genomic DNA and 10 μM of each primer. We used 10 mM dNTPs (dGTP/7-deaza-dGTP = 1/1), 5% dimethyl sulfoxide, 10 μM of each primer, and a reaction mixture of 0.4 U of Q5^®^ High-Fidelity DNA Polymerase and 4 μL of Q5 High GC Enhancer (Biolabs England, Tokyo, Japan). A programmable thermal cycler was used, with an initial denaturation step at 98 °C for 30 s, 45 cycles (98 °C for 10 s, 58 °C for 30 s, and 72 °C for 30 s), and a final extension step of 72 °C for 2 min. The reaction products were electrophoresed in 2% agarose gel (Takara, Tokyo, Japan) with ethidium bromide and visualized by ultraviolet illumination. After PCR amplification, the genotyping was performed by agarose gel electrophoresis. The 5-HTTLPR length variation was defined by two variable nucleotide tandem repeat elements. The l/l genotype had a band of 512 bp, the s/l (hetero) genotype had bands of 469 and 512 bp, and the s/s genotype had a band of 469 bp.

#### 2.4.2. COMT

Thereafter, as described by Lajin et al. [40], PCR amplification was conducted using the COMT primers F/R 5′-CGAGGCTCATCACCATCGAGATC-3′ and 5′-CTGACAACGGGTCAGGAATGCA-3′. The reaction mixture consisted of a total volume of 20 μL, including 5–10 ng of genomic DNA, 10 μM of each primer, 10 mM of dNTP mix, 1 × Taq buffer and 0.2 μL of Taq DNA polymerase (Biolabs England, Tokyo, Japan), 0.4 U of Q5^®^ High-Fidelity DNA Polymerase, and 4 μL of Q5 High GC Enhancer (Biolabs England, Tokyo, Japan). PCR amplification was performed using a programmable thermal cycler. The initial denaturation step for COMT was conducted at 98 °C for 30 s, followed by 45 cycles (98 °C for 10 s, 58 °C for 30 s, and 72 °C for 30 s), and extension at 72 °C for 2 min. After that, we mixed the whole volume of the unpurified PCR product with 1 μL of NlaIII (Biolabs England, Tokyo, Japan) and 2 μL of 10 × CutSmart Buffer. Digestion was ensured to be complete following incubation at 37 °C for 20 min. It showed three bands in heterozygotes (108, 72, and 36 bp). Finally, the reaction products were electrophoresed through 3% agarose gel (Takara, Tokyo, Japan) with ethidium bromide and visualized by ultraviolet illumination. After digestion, agarose gel electrophoresis showed a single 108 bp band in Val homozygotes. In Met homozygotes, second bands were produced (72 and 36 bp).

### 2.5. Statistical Analyses

Statistical analysis was performed using the Mann–Whitney U test, Kruskal–Wallis test, and repeated-measure designs by ANOVA and MANOVA. When significant main effects and interactions were found, they were followed by the Bonferroni’s multiple comparison method. A *p* value of less than 0.05 (*p* < 0.05) was considered significant. The statistical software used for the analyses was SPSS for Windows (24.0J, IBM Japan, Chuo-ku, Japan).

## 3. Results

### 3.1. 5-HTTLPR and COMT Genotype Frequencies

The frequencies of the 5-HTTLPR genotypes s/s, s/l, and l/l were 22, 12, and 0, respectively, and those of COMT Met/Met, Met/Val, and Val/Val were 7, 23, and 4, respectively. The breakdown is shown in Table 1. There was no difference in the distribution of 5-HTTLPR. However, a difference existed in one of the COMT genotypes between the Y-Elite and Pro dancers. 

### 3.2. Relationship Between the STAI and 5-HTTLPR or COMT Genotypes 

The results showed that the trait anxiety scores of the Y-Elite dancers were higher than those of the Pro dancers by the Mann–Whitney U test (usual: *p* < 0.036; pre-competition: *p* < 0.005). Furthermore, especially before a competition, where dancers experience increased stress, the trait anxiety score of the Y-Elite dancers was higher than that of the Pro dancers for each genotype group by the Kruskal–Wallis test (5-HTTLPR: *p* < 0.027; COMT: *p* < 0.019) (Table 2). 

We next surveyed the repeated-measures ANOVA and MANOVA (5-HTTLPR × COMT × Pro/Y-Elite) results for the STAI on a usual practice day and one week before competition day. However, no significant association was observed between these genotypes and the STAI across all conditions.

### 3.3. Relationship Between the BRUMS and 5-HTTLPR or COMT Genotypes 

**Figure 1:**
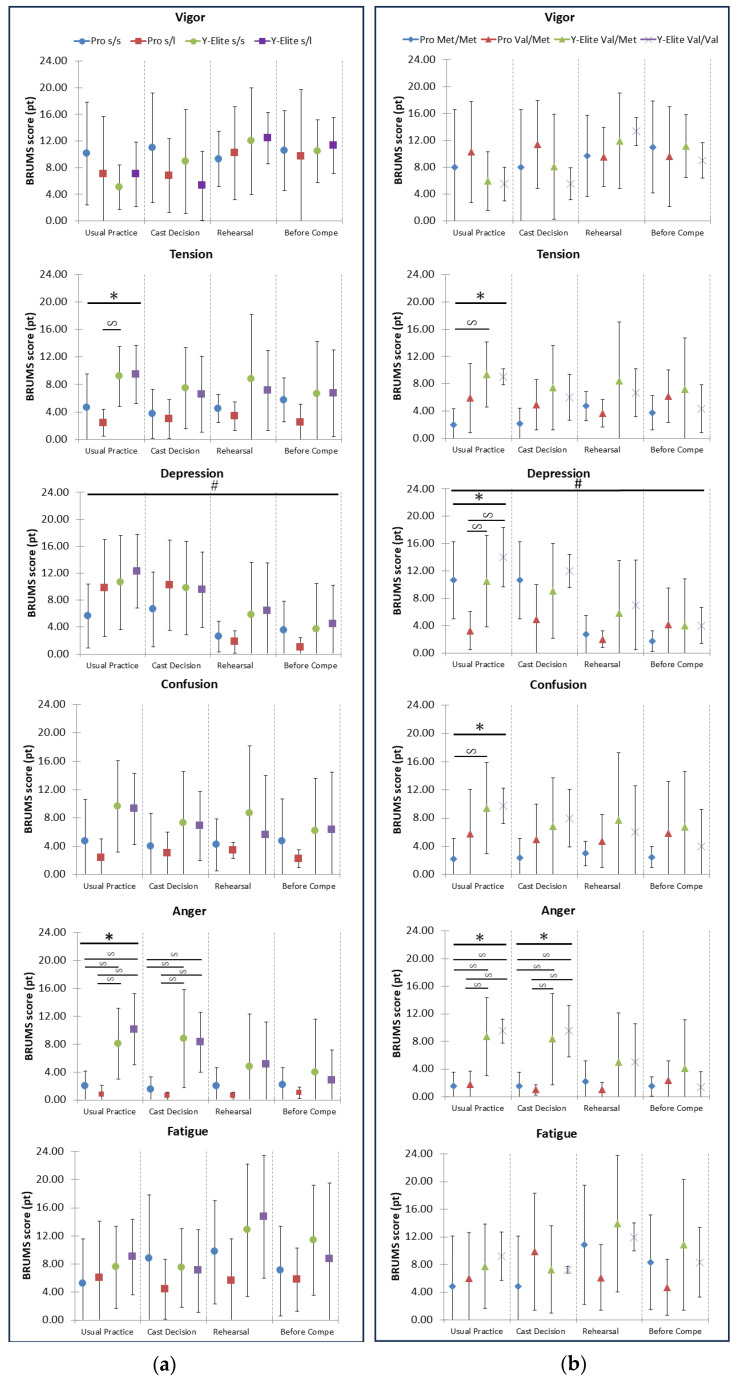
BRUMS on four different conditions (average ± SD): (**a**) the 5-HTTLPR group; (**b**) the COMT group. Note: BRUMS: Brunel Mood Scales; 5-HTTLPR: the serotonin transporter gene polymorphism region; COMT: the Catechol-O-methyltransferase protein; s: the short variation allele; l: the long variation allele; Val: Val (G) allele carriers; Met: Met (A) allele carriers; Usual Practice: usual practice day; Cast Decision: cast decision day; Rehearsal: rehearsal day; Before Compe.: one week before competition day; Genome groups’ differences through four conditions: *p* < 0.05 #. Four groups’ differences in each condition: *p* < 0.05 *. Two groups’ differences in each condition: *p* < 0.05 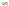. Explanation of symbols and colors: circles (●) are marked s/s, squares (■) are marked s/l, rhombuses (◆) are marked Met/Met, triangles (▲) are marked Val/Met and crosses (×) are marked Val/Val according to genotype. Light blue and red indicate Pro group; yellow-green and purple indicate Y-Elite group.

Regarding mood in the BRUMS, in the 5HTTLPR × COMT × Pro/Y-Elite repeated-measures MANOVA, six mood scales were significantly different or tended to be different through four conditions (Wilks’ λ; four conditions: *p* < 0.004, 5HTTLPR: *p* = 0.070Δ, COMT: *p* < 0.012, Pro/Y-Elite: *p* < 0.005, and 5HTTLPR × Pro/Y-Elite: *p* = 0.063Δ). After that, genome groups were broken down by “Pro” and “Y-Elite,” and the genome groups were analyzed and visualized separately (Figure 1).

#### 3.3.1. In 5-HTTLPR Genotype Group

When examined by factors, in the 5-HTTLPR group (Figure 1a), there was no difference through the four stress conditions, but there was a main effect on the “Depression” scale by the subtest (Mauchly’s W; F (3,66) = 2.821, *p* = 0.047). But there were no significant interactions between the 5-HTTLPR genotype and the Pro/Y-Elite groups. On a usual practice day, the Tension and Anger scores for the Pro dancers were significantly lower than those for the Y-Elite dancers (ps < 0.05). Anger scores were also lower for the Pro dancers on the cast decision day (ps < 0.05).

Concerning average Depression scores, the Y-Elite level with the hetero (s/l genotype) and short (s/s genotype) genotypes showed higher scores than the Pro level. Therefore, we can consider that Pro dancers tend to be more stable than Y-Elite dancers in negative scales.

#### 3.3.2. In COMT Genotype Group

In the COMT group (Figure 1b), there was a significant difference through the four stress conditions (Wilks’ λ; *p* < 0.015), and there was especially a main effect on the “Depression” scale (Mauchly’s W; F (6,66) = 4.475, *p* < 0.001). But there were no calculated interactions between the COMT genotype and the Pro/Y-Elite groups through the four conditions. On a usual practice day, Tension, Depression, Confusion, and Anger scores for the Pro dancers were significantly lower than those for the Y-Elite dancers (ps < 0.05). Anger scores were also lower for the Pro dancers on the cast decision day (ps < 0.05).

The Y-Elite participants with both Val/Met and Val/Val genotypes exhibited higher average Depression scores compared to the Pro participants with the Val/Met genotype (*p* < 0.037; *p* < 0.021). This difference was significant among the four groups on a usual practice day (*p* < 0.01). 

### 3.4. Relationship Between the BRUMS and Pain Feeling in 5-HTTLPR or COMT Genotypes

Next, we evaluated the relationship between mood and pain feeling; in the 5HTTLPR × COMT × Pro/Y-Elite repeated-measures MANOVA, the six mood scales were significantly different through the four stress conditions (Wilks’ λ; four conditions: *p* < 0.014, 5-HTTLPR: *p* < 0.019, COMT: *p* < 0.038, Pro/Y-Elite: *p* < 0.006, Pain: *p* < 0.041, 5-HTTLPR × Pro/Y-Elite: *p* < 0.014, 5-HTTLPR × Pain: *p* < 0.020, and COMT × Pain: *p* < 0.046).

Regarding the relationship between the self-report of pain and injury and its interaction with mood for the six scales, there tended to be non-significant differences in the COMT group and the Pro/Y-Elite groups across the four conditions (Wilks’ λ; COMT: *p* = 0.099Δ and Pro/Y-Elite: *p* = 0.074Δ). There was a main effect only on the “Depression” scale in COMT (Mauchly’s W; F (6,54) = 2.643, *p* < 0.026). Youth elite dancers’ self-report of feeling pain increased as they neared competition. However, in the medical checks, in contrast to the painful feeling reported by them, their injuries decreased through the four conditions. In the Pro dancers, the moods differed significantly within Pain feeling × COMT, although their injuries sustained (see Table 3).

## 4. Discussion

This study investigated the relationship between candidate genes, 5-HTT and COMT, and anxiety when ballet dancers with pain are placed under stress. We found that 5-HTTLPR and COMT played a crucial role in the background of anxiety and mood over a long period from daily practice to performance day. We think that Y-Elite dancers’ anxiety is more likely due to occupational (environmental) factors and personal stress than genetic factors. This result contrasts with the results from other studies, which showed that the Val allele of the COMT gene has a strong COMT activity and reduced pain sensitivity [7,12]. A reason for this inconsistency might be that Pro ballet dancers are predicted by their lower neuroticism and mood scores and their better adaptation to stress than Y-Elite dancers. The 5-HTTLPR and COMT genes may be influencing the sensitivity to the environment. Therefore, youth elite ballet dancers should understand the relationship between pain and physical activity from an early stage.

The short Met/Met COMT genotype appears to affect large fluctuations in Pro dancers. Youth elites are more likely to be anxious due to occupational and personal stress than genetic factors. In other words, the BRUMS scores increase/decrease with the short Met/Met genotype and fluctuate easily according to stress. However, we could not determine this in detail across the four conditions because the COMT genotypes in the Pro and Y-Elite dancers were unbalanced in this study, as some of the dancers were absent due to influenza on the measurement day. 

Youth elite dancers often felt pain and stress during the casting or choreography learning periods. However, their injury pain increased/worsened as the competition neared. Their self-reports showed increased pain from injury near the competition. Half of the Y-Elite dancers had feelings of pain even after the rehearsal period and the period approaching the performance, as can be seen in Table 3. Moreover, the Y-Elite dancers appeared to be unable to maintain their physical health, probably because they were practicing ballet too much. Although they felt a painful sensation, the number of injuries found in the medical examinations was less than the number of pain complaints. Some types of pain caused by sports injuries that cannot be found in medical checks may be manifested as subjective pain feelings when stressed [15,18,39]. 

It is essential to understand the mechanisms of pain and pain relief before the worsening of trauma or disability. Therefore, it is crucial for ballet dancers to undergo medical checks [9,14,41,42,43,44], always acknowledging the significant impact of pain memory on subsequent performance. Intense nociceptive, psychogenic (psychological and social), and neuropathic pain are likely to occur as a result of sports injuries. If nociceptive pain persists for an extended period, the influence of neuropathic pain becomes more pronounced. Therefore, initial pain treatment is essential [45,46,47,48]. Pro dancers perceive pain as positive performance pain; youth elites perceive pain as negative injury pain [9]. 

A limitation of our research was that we did not investigate in detail whether the pain was caused by an injury. However, from the results of the genomic polymorphism analysis, it seemed that some emotional control processes influenced the subjective evaluation. Still, it appeared that the environmental factors of being Pro and Y-Elite dancers were strongly expressed. Nevertheless, there appears to be some relationship between negative mood and pain in youth elites. 

By explaining the interactions between loci, it becomes possible to understand the complex genetic factors that have a more profound impact on psychosocial development, and, consequently, on mental health, than individual loci alone [44]. In future research, it will be necessary to expand the sample size to conduct more detailed investigations. Previous studies suggest that the interplay between temperament (genetic factors) and personality contributes to individuals’ susceptibility to performance impairment (choking under pressure) in occupational settings [49,50,51]. However, instead of being viewed as an outcome, it is considered that this susceptibility arises from disparities in accumulated experience and occupational demands.

## 5. Conclusions

This study explored the relationship of candidate genes for pain with anxiety and mood. Although anxiety may be affected by occupational experience and environmental factors, the results showed that 5-HTTLPR and COMT play a crucial role in affecting dancers’ anxiety and mood in the presence of pain.

We found associations between depressed affect and polymorphisms with anxiety-related traits. Although hereditary factors may have been present, the results from the subjective questionnaires indicated that occupational factors strongly influenced mood. Specifically, youth elite dancers often felt stressed and pained during the practice period. However, their injury pain increased significantly as the competition approached. Pro ballet dancers were more accustomed to pain and could thus observe pain features more objectively [48]. It is likely that this finding is due to their lower levels of neuroticism and mood scores, as well as their superior ability to adapt to stress compared to youth elite ballet dancers. The pain appears to be accepted as a necessity and often ignored; dancing appears to continue despite the pain [9]. The 5-HTTLPR and COMT genes may be influencing the sensitivity to the environment. This process may lead to changes in character and behavior. 

Pro dancers view pain from a more stable perspective. In addition to mental stability, knowing the reason for their pain and learning how to cope with it is something dancers studying ballet as students or in technical schools should be aware of as early as possible, preferably before they start dancing professionally.

## Figures and Tables

**Table 1:** Frequencies of the 5-HTTLPR and COMT genotypes.

Genotype		Pro (*n* = 16)		Y-Elite (*n* = 18)		All (*n* = 34)	
		*n*	(%)	*n*	(%)	*n*	(%)
5-HTTLPR	s/s	11	(68.8)	11	(61.1)	22	(64.7)
	s/l	5	(31.3)	7	(38.9)	12	(35.3)
	l/l	0	(0.0)	0	(0.0)	0	(0.0)
COMT	Met/Met	7	(43.8)	0	(0.0)	7	(20.6)
	Val/Met	9	(56.2)	14	(77.8)	23	(67.6)
	Val/Val	0	(0.0)	4	(22.2)	4	(11.8)

Note: 5-HTTLPR: the serotonin transporter gene polymorphism region; COMT: the Catechol-O-methyltransferase protein; s: the short variation allele; l: the long variation allele; Val: Val (G) allele carriers; Met: Met (A) allele carries.

**Table 2:** STAI according to the classification of the 5-HTTLPR and COMT genes.

(A) Trait Anxiety						
**Usual**		**Pro (*n* = 16)**		**Y-** **Elite (*n* = 16)**		***p***
		Average	SD	Average	SD	**0.035** #
5-HTTLPR	s/s	43.82	11.06	50.30	10.85	
	s/l	42.60	8.44	53.17	6.24	n.s. †
	l/l	-	-	-	-	
COMT	Met/Met	42.13	11.66	-	-	
	Val/Met	44.75	8.75	50.77	10.09	n.s. †
	Val/Val	-	-	54.00	4.36	
**Before Comp.**		**Pro (*n* = 16)**		**Y-** **Elite (*n* = 13)**		***p***
		Average	SD	Average	SD	**0.004** #
5-HTTLPR	s/s	43.09	9.84	51.38	9.23	
	s/l	40.80	6.91	57.60	9.94	**0.026** †
	l/l	-	-	-	-	
COMT	Met/Met	43.38	8.99	-	-	
	Val/Met	41.38	9.23	51.91	9.29	**0.018** †
	Val/Val	-	-	64.00	0.00	
**(B) State Anxiety**						
**Usual**		**Pro (*n* = 16)**		**Y-** **Elite (*n* = 16)**		***p***
		Average	SD	Average	SD	n.s. #
5-HTTLPR	s/s	46.36	13.40	47.40	10.22	
	s/l	41.80	3.35	45.75	9.36	n.s. †
	l/l	-	-	-	-	
COMT	Met/Met	43.88	12.68	-	-	
	Val/Met	46.00	10.49	45.60	11.05	n.s. †
	Val/Val	-	-	50.25	4.11	
**Before Comp.**		**Pro (*n* = 16)**		**Y-** **Elite (*n* = 13)**		***p***
		Average	SD	Average	SD	n.s. #
5-HTTLPR	s/s	49.45	9.61	44.50	8.80	
	s/l	40.20	10.43	47.40	6.50	n.s. †
	l/l	-	-	-	-	
COMT	Met/Met	44.13	10.63	-	-	
	Val/Met	49.00	10.46	45.73	8.57	n.s. †
	Val/Val	-	-	45.00	1.41	

Note. STAI: State-Trait Anxiety Inventory; 5-HTTLPR: the serotonin transporter gene polymorphism region; COMT: the Catechol-O-methyltransferase protein; s: the short variation allele; l: the long variation allele; Val: Val (G) allele carriers; Met: Met (A) allele carriers; Usual: usual practice day; Before Comp.: one week before competition day; SD: standard deviation. n.s.: no significant; Pro vs. Y-Elite: *p* < 0.05 #; Among group in genotypes: *p* < 0.05 †.

**Table 3:** Pain feeling and injury ratio under the four conditions.

Group	Pre	Usual P.		Cast Dec.		Rehear.		Before Comp.	
		1–2 w	3–4 w	1–2 w	3–4 w	1–2 w	3–4 w	1–2 w	3–4 w
Pain † (Pro)	25.0%	37.5%		22.2%		**50.0%**		38.9%	
* (Y-Elite)	33.3%	27.8%		38.9%		44.4%		**50.0%**	
IR (Pro)	-	0.0	0.0	0.0	0.0	0.1	0.1	0.1	0.1
(Y-Elite)	-	1.2	0.9	0.6	0.9	0.5	0.4	0.3	0.4

Note. Pain: the subjective pain feeling; w: week; Usual: usual practice day; Cast Dec.: cast decision day; Rehear.: rehearsal day; Before Comp.: one week before competition day; * 5-HTTLPR or † COMT × Pain feeling × BRUMS: *p* < 0.05; IR: Incident Ratio.

## Data Availability

The data associated with the paper are not publicly available due to them containing information that could compromise research participants’ privacy/consent, but may be obtained from the corresponding author on reasonable request.

## References

[1] Caine D., Goodwin B.J., Caine C.G., Bergeron G. (2015). Epidemiological review of injury in pre-professional ballet dancers. J. Dance Med. Sci..

[2] Girard J., Koenig K., Village D. (2015). The effect of strength and plyometric training on functional dance performance in elite ballet and modern dancers. Phys. Ther. Rev..

[3] (2021). The activity demands and physiological responses observed in professional ballet: A systematic review. J. Sport Exerc. Sci..

[4] Twitchett E.A., Koutedakis Y., Wyon M.A. (2009). Physiological fitness and professional classical ballet performance: A brief review. J. Strength Cond. Res..

[5] Mainwaring L.M., Finney C. (2017). Psychological risk factors and outcomes of dance injury: A systematic review. J. Dance Med. Sci..

[6] Evans R.I.E.R.W., I Evans R., Carvajal S., Perry S. (1996). A survey of injuries among Broadway performers. Am. J. Public Health.

[7] Fuller M., Hunt A., Minett G. (2020). Injuries during transition periods across the year in pre-professional and professional ballet and contemporary dancers: A systematic review and meta-analysis. Phys. Ther. Sport.

[8] Kozai A.C., Twitchett E., Morgan S., Wyon M.A. (2020). Workload intensity and rest periods in professional ballet: Connotations for injury. Int. J. Sports Med..

[9] Ryan A.J., Stephens R.E., Ryan A.J., Stephens R.E. (1987). The epidemiology of dance injuries. Dance Medicine: A Comprehensive Guide.

[10] Twitchett E., Angioi M., Koutedakis Y., Wyon M. (2010). The demands of a working day among female professional ballet dancers. J. Dance Med. Sci..

[11] Hernandez B.M. (2013). Addressing Occupational Stress in Dancers. J. Phys. Educ. Recreat. Dance.

[12] Konijn E.A., Wilson G.D. (1991). What’s on between the actor and his audience? Empirical analysis of emotion processes in the theatre. Psychology and Performing Arts.

[13] Yatabe K., Yui N., Kasuya S., Fujiya H., Tateishi K., Terawaki F., Yoshida A., Yoshioka H., Terauchi K., Miyano H. (2014). Anxiety and mood among ballet dancers: A pilot study on effects of a medical approach involving periodic intervention. Ann. Sport Med. Res..

[14] Ostwald P.F., Baron B.C., Byl N.M., Wilson F.R. (1994). Wilson, Performing arts medicine. West. J. Med..

[15] Marchant-Haycox S.E., Wilson G.D. (1992). Personality and stress in performing artists. Pers. Individ. Differ..

[16] Sprangers M.A.G., Thong M.S.Y., Bartels M., Barsevick A., Ordoñana J., Shi Q., Wang X.S., Klepstad P., Wierenga E.A., Singh J.A. (2014). Biological pathways, candidate genes, and molecular markers associated with quality-of-life domains: An update. Qual. Life Res..

[17] Lesch K.-P., Bengel D., Heils A., Sabol S.Z., Greenberg B.D., Petri S., Benjamin J., Müller C.R., Hamer D.H., Murphy D.L. (1996). Association of anxiety-related traits with a polymorphism in the serotonin transporter gene regulatory region. Science.

[18] Bachner-Melman R., Dina C., Zohar A.H., Constantini N., Lerer E., Hoch S., Sella S., Nemanov L., Gritsenko I., Lichtenberg P. (2005). AVPR1a and SLC6A4 Gene polymorphisms are associated with creative dance performance. PLoS Genet..

[19] Miller R., Wankerl M., Stalder T., Kirschbaum C., Alexander N. (2013). The serotonin transporter gene-linked polymorphic region (5-HTTLPR) and cortisol stress reactivity: A meta-analysis. Mol. Psychiatry.

[20] Gelernter J., Kranzler H., Cubells J.F. (1997). Serotonin transporter protein (SLC6A4) allele and haplotype frequencies and linkage disequilibria in African- and European-American and Japanese populations and in alcohol-dependent subjects. Hum. Genet..

[21] Nakamura T., Muramatsu T., Ono Y., Matsushita S., Higuchi S., Mizushima H., Yoshimura K., Kanba S., Asai M. (1997). Serotonin transporter gene regulatory region polymorphism and anxiety-related traits in the Japanese. Am. J. Med. Genet..

[22] Yatabe K., Kumai T., Fujiya H., Yui N., Kasuya S., Murofushi Y., Tateishi K., Terawaki F., Kobayashi H., Uchino A. (2016). Effects of serotonin transporter gene polymorphism on mood during the period before the competition in Japanese ballet dancers. Integr. Mol. Med..

[23] Lee L.O., Prescott C.A. (2014). Association of the catechol-O-methyltransferase val158met polymorphism and anxiety-related traits: A meta-analysis. Psychiatr. Genet..

[24] Lajin B., Alachkar A., Hamzeh A., Michati R., Alhaj H. (2011). No association between Val158Met of the COMT gene and susceptibility to schizophrenia in the Syrian population. N. Am. J. Med. Sci..

[25] Montag C., Jurkiewicz M., Reuter M. (2012). The role of the catechol-O-methyltransferase (COMT) gene in personality and related psychopathological disorders. CNS Neurol. Disord. Drug Targets.

[26] Olsson C.A., Anney R.J., Lotfi-Miri M., Byrnes G.B., Williamson R., Patton G.C. (2005). Association between the COMT Val158Met polymorphism and propensity to anxiety in an Australian population-based longitudinal study of adolescent health. Psychiatr. Genet..

[27] Zubieta J.-K., Heitzeg M.M., Smith Y.R., Bueller J.A., Xu K., Xu Y., Koeppe R.A., Stohler C.S., Goldman D. (2003). COMT *val^158^met* genotype affects µ-opioid neurotransmitter responses to a pain stressor. Science.

[28] Spielberger C.D. (1991). Manual for the State-Trait Anger-Expression Inventory.

[29] Spielberger C.D., Form Y., Spielberger C.D., Gorsuch R.L., Lushene R.E., Vagg P.R., Jacobs G.A. (1983). Manual for the State–Trait Anxiety Inventory.

[30] Spielberger C.D., Mizuguchi T., Shimonaka Y., Nakazato K., Sankyoubou K. (1991). The Japanese Version of STAI.

[31] Terry P.C., Lane A.M. (2003). User Guide for the Brunel Mood Scale (BRUMS).

[32] Terry P.C., Parsons-Smith R.L. (2021). Mood profiling for sustainable mental health among athletes. Sustainability.

[33] Terry P.C., Lane A.M., Lane H.J., Keohane L. (1999). Development and validation of a mood measure for adolescents. J. Sports Sci..

[34] Terry P., Lane A., Fogarty G. (2003). Construct validity of the Profile of Mood States—Adolescents for use with adults. Psychol. Sport Exerc..

[35] Beedie C.J., Terry P.C., Lane A.M. (2000). The profile of mood states and athletic performance: Two meta-analyses. J. Appl. Sport Psychol..

[36] Terry P.C., Parsons-Smith R.L., King R., Terry V.R. (2021). Influence of sex, age, and education on mood profile clusters. PLoS ONE.

[37] Yatabe K., Oyama T., Fujiya H., Kato H., Seki H., Kohno T. (2006). Development and validation of the preliminary Japanese version of the profile of mood states for adolescents, St. Marianna. Med. J..

[38] Yatabe K., Oyama T. (2007). Reliability and validity of the Profile of Mood State for adolescent: The second report. Proceedings of the Annual Convention of the Japanese Psychological Association (Nihon Shinri Gakkai Taikai Happyo Ronbunsyu).

[39] Pieper S., Out D., Bakermans-Kranenburg M.J., van Ijzendoorn M.H. (2011). Behavioral and molecular genetics of dissociation: The role of the serotonin transporter gene promoter polymorphism (5-HTTLPR). J. Trauma. Stress.

[40] Lajin B., Alachkar A. (2011). Detection of catechol-O-methyltransferase (COMT) Val158Met Polymorphism by a New Optimized PCR-RFLP Method. Am. J. Biomed. Sci..

[41] Byhring S., Bø K. (2002). Musculoskeletal injuries in the Norwegian National Ballet: A prospective cohort study. Scand. J. Med. Sci. Sports.

[42] Miller C. (2006). Dance medicine: Current concepts. Phys. Med. Rehabil. Clin. N. Am..

[43] Nilsson C., Leanderson J., Wykman A., Strender L.-E. (2001). The injury panorama in a Swedish professional ballet company. Knee Surgery Sports Traumatol. Arthrosc..

[44] Solomon R., Solomon J., Micheli L.J., McGray E. (1999). The cost of injuries in a professional ballet company: A five-year study. Med. Probl. Perform. Artist..

[45] Hincapié C.A., Morton E.J., Cassidy J.D. (2008). Musculoskeletal injuries and pain in dancers: A systematic review. Arch. Phy. Med. Rehab..

[46] Lampe J., Borgetto B., Groneberg D.A., Wanke E.M. (2018). Prevalence, localization, perception and management of pain in dance: An overview. Scand. J. Pain.

[47] Olsson C.A., Byrnes G.B., Anney R.J.L., Collins V., Hemphill S.A., Williamson R., Patton G.C. (2007). COMT Val^158^Met and 5HTTLPR functional loci interact to predict persistence of anxiety across adolescence: Results from the Victorian Adolescent Health Cohort Study. Genes Brain Behav..

[48] Tajet-Foxell B., Rose F.D. (1995). Pain and pain tolerance in professional ballet dancers. Br. J. Sports Med..

[49] Baumeister R.F. (1984). Choking under pressure: Self-consciousness and paradoxical effects of incentives on skillful performance. J. Pers. Soc. Psychol..

[50] Murayama T., Sekiya H., Tanaka Y. (2010). Factor analysis of the mechanisms underlying ‘choking under pressure’ in sports. Asian J. Exerc. Sport. Sci..

[51] Wang J., Marchant D., Morris T., Gibbs P. (2004). Self-consciousness and trait anxiety as predictors of choking in sport. J. Sci. Med. Sport.

